# Meig’s Obstetrics

**Published:** 1849-06

**Authors:** 


### MEIGS' OBSTETRICS.

We have had for some time a copy of this valuable treatise on our table, but hitherto have been unable to give it the full notice to which it is entitled. We hope to be able to do so in our next number. In the meantime we cordially recommend it to our readers. Several other books have been received from the publishers, which we shall embrace the earliest opportunity to notice. One of these is Kirkes' & Paget's Physiology.

